# Horseshoe Abscess as a Rare Complication After Transanal Hemorrhoid Dearterialization With Mucopexy: A Case Report

**DOI:** 10.7759/cureus.51195

**Published:** 2023-12-27

**Authors:** Yuki Nakao, Heather Reeves, David A Denning, James Martin

**Affiliations:** 1 General Surgery, Marshall University Joan C. Edwards School of Medicine, Huntington, USA; 2 General Surgery, Huntington VA (Veterans Affairs) Medical Center, Huntington, USA

**Keywords:** postoperative complication, mucopexy, horseshoe abscess, transanal hemorrhoidal dearterialization, hemorrhoid

## Abstract

Transanal hemorrhoidal dearterialization (THD) is a minimally invasive procedure that has gained popularity as a treatment for symptomatic hemorrhoids. It involves ligating the arterial blood supply to the hemorrhoidal plexus. Compared to conventional ligation or resection, THD is associated with less postoperative bleeding and pain, allowing for same-day surgery discharge. Horseshoe abscess is a rare but known complication of anorectal surgery, characterized by an abscess that extends around the anal canal, often involving the ischiorectal fossa and adjacent structures. Although horseshoe abscesses have been reported after various anorectal surgeries, including hemorrhoidectomy, their occurrence following THD has not been well-documented in the literature.

A 72-year-old male underwent THD for rectal prolapse with internal hemorrhoids and presented to the hospital on postoperative day 6 with severe rectal pain. A computed tomography (CT) scan revealed a large complex horseshoe perirectal abscess with fluid and air and significant rectal wall thickening. A rectal examination under anesthesia confirmed the presence of purulent drainage from the anus, and surgical drainage of the abscess was performed. The patient received antibiotics and analgesics and experienced a favorable recovery.

The exact pathophysiology of a horseshoe abscess following THD remains unclear, and the incidence and risk factors associated with this complication are not well-established. Moreover, there has yet to be a consensus on the optimal management of horseshoe abscesses after THD, whether through surgical or medical approaches. This case emphasizes the importance of considering horseshoe abscess as a potential complication of THD and highlights the need for further research to understand better its incidence, risk factors, and optimal management strategies.

## Introduction

Hemorrhoidal disease is a common condition that affects approximately 4.4% of the population in the United States [1]. Transanal hemorrhoidal dearterialization (THD) is a minimally invasive procedure that has become increasingly popular as a treatment for symptomatic hemorrhoids. This technique involves the ligation of the arterial blood supply to the hemorrhoidal plexus, leading to a reduction in bleeding, prolapse, and pain. Despite its advantages, THD is not without risks. Complications associated with THD include bleeding, infection, anal fistula, and urinary retention [2].

Horseshoe abscess is a rare but well-known complication of anorectal surgery. It is characterized by an abscess that extends around the circumference of the anal canal, often involving the ischiorectal fossa and other adjacent structures [3]. Although horseshoe abscess has been reported after several anorectal surgeries, including hemorrhoidectomy, its occurrence after THD has not been well documented in the literature.

The exact pathophysiology of horseshoe abscess after THD is not fully understood. The incidence of horseshoe abscess after THD is not well established, and its risk factors are unclear. Furthermore, the optimal management of horseshoe abscess after THD is not well defined, and there is no consensus on the best surgical or medical approach. In this report, we describe a case of a patient who came to our hospital six days after THD surgery with a complaint of anorectal pain, and whose CT scan showed a horseshoe abscess.

This article will be presented as a poster at the 2024 SAGES (Society of American Gastrointestinal and Endoscopic Surgeons) Annual Meeting, Cleveland, OH, on April 17-20, 2024.

## Case presentation

A 72-year-old male, with a medical history of chronic obstructive pulmonary disease, and who was a current smoker, was referred for anal bleeding, which was associated with constipation over the past 10 years. His physical examination showed a stage IV rectal prolapse with internal hemorrhoid. The rectal examination under anesthesia with the repair of the rectal prolapse with transanal hemorrhoidal dearterialization was performed electively. With the patient under general anesthesia and lying in the lithotomy position, a folded 4x4 gauze was placed into the rectum and withdrawn slowly to simulate the passage of stool. The rectal mucosa did prolapse and did not automatically return to the rectum, so it was classified as a grade IV rectal prolapse. Dilated internal hemorrhoids did accompany this prolapse. Examination of the anus and perineum first revealed no ano-dermal skin lesions. A digital examination was then done, and the anal tone was found to be normal. There were no visible or palpable mucosal irregularities of the anoderm or the anal canal. The anal sphincter was uniform in its thickness without palpable masses or nodules. There was no anal fissure or scarring. There were engorged hemorrhoidal cushions from the 1 o’clock to the 5 o’clock positions. We used the Doppler ultrasound probe to identify the six terminal branches of the superior rectal artery (located at 1, 3, 5, 7, 9, and 11 o’clock). Each was identified and ligated with a figure 8 stitch of a coated Vicryl suture. With the same suture, a mucopexy was performed by a continuous running suture, making 2-4 mucosal stitches ending at least 5 mm above the dentate line. After the mucopexy was completed, the anus and peri-rectum were injected in all four quadrants with a 1% xylocaine and 0.5% bupivacaine mixture for analgesia. The patient was discharged home on the same day.

His pain was well controlled postoperatively; however, he started experiencing significantly increased rectal pain on postoperative day (POD) 5. He then presented to the ER on POD 6. He denied any nausea, vomiting, fevers, or chills. He noticed some whitish-brown discharge from the area when passing the flatus. He continued to have small bowel movements, but claimed his last decent bowel movements were three days ago. He had been using sitz baths and narcotics to control the pain. A physical examination revealed a heart rate of 76 beats/min, regular rhythm, blood pressure of 100/63 mmHg, a respiratory rate of 22 breaths/min, temperature 37.0℃, and oxygen saturation of 97% on room air. The area of erythematous anal swelling at the 6 o’clock position was severely tender to palpation with no apparent discharge or active bleeding. A digital examination could not be performed due to a pain level of 10/10.

**Figure 1:**
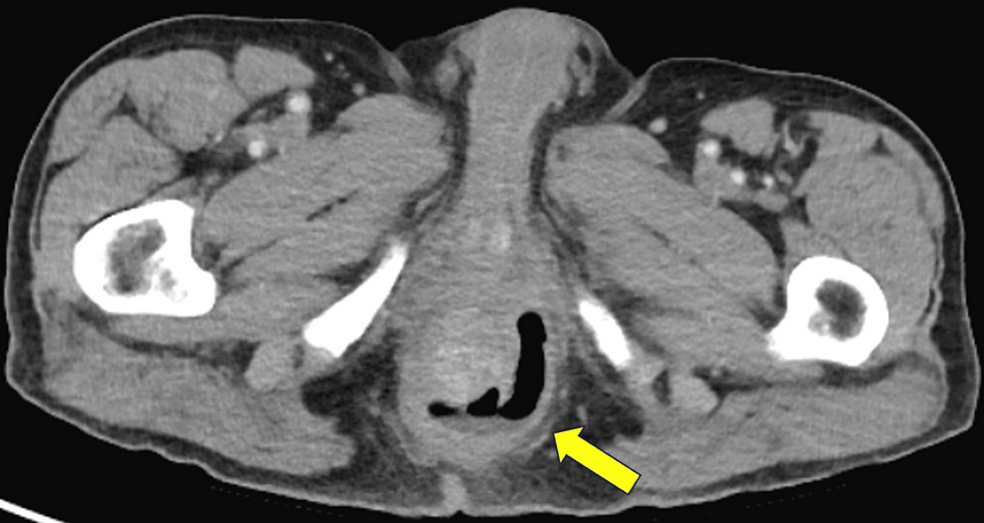
CT of the abdomen/pelvis with IV contrast A large complex horseshoe perirectal abscess containing fluid and air with marked thickening of the rectum is seen.

Laboratory tests revealed a hemoglobin level of 14.6 g/dL (normal 14-18 g/dL), white cell count of 18.6 x 10^9^/L (normal 4.5-11 x 10^9^/L), lactic acid level of 1.2 mmol/L (normal <2.0 mmol/L), and a sodium level of 127 mEq/L (normal 136-142 mEq/L). Other laboratory data were within normal limits. EKG showed a normal sinus rhythm with no ST changes. Abdominal/pelvic CT imaging was performed that showed a large complex horseshoe perirectal abscess containing fluid and air with marked thickening of the rectum (Figure 1).

The patient underwent a rectal examination under anesthesia, which revealed purulent drainage from the anus. No external openings were found. Examination of the perineum revealed swelling and a bulge localized to the left side of the midline. Two incisions were made at the 1 and 5 o’clock positions to evacuate a suspected perirectal fluid collection. The seropurulent drainage was evacuated from the space and an opening into the rectal vault was identified. The mucopexy suture at the 5 o’clock position was loose and thus it was removed. Two half-inch Penrose drains were placed along two fistulous tracts as setons.

The patient had no postoperative complications. He was able to tolerate a regular diet and was discharged home on postoperative day 7.

## Discussion

This case report described the transanal hemorrhoidal dearterialization technique, first introduced in 1995 by Morinaga et al. as a surgical treatment for internal hemorrhoids [1]. This technique works by using a specially developed anoscopic combined with a Doppler transducer to identify the hemorrhoidal arteries, which originate from the superior rectal artery, 2-3 cm above the pectinate line. Once the superior rectal arteries are identified through the Doppler, a suture ligation is performed to cut off the blood supply to the swollen hemorrhoids. This prevents any further arterial blood from filling up the hemorrhoid, while the venous outflow remains to let the hemorrhoid shrink. If the hemorrhoid is prolapsed, the surgeon will perform a lift, called a hemorrhoidopexy, which restores the protruding tissue into its correct position. Unlike other types of hemorrhoid treatments, THD involves no cutting, and only restricting the blood vessels. THD is recommended for grade II and III hemorrhoidal disease. For grade IV cases, where the prolapse cannot be reduced into the rectum, conventional hemorrhoidectomy is a more suitable option. It is important to note that the presence of a complete rectal prolapse is considered a contraindication to THD [1]. This technique was developed specifically to reduce postoperative complications, such as pain, and has undergone a series of refinements that have broadened its indications and improved its outcomes. The surgical technique is not standardized, and the results for advanced hemorrhoidal disease are controversial [4]. The success rate for THD varies based on the severity of hemorrhoidal disease and the specific surgical technique employed. In one study, considering reoperations and assessing patients at the final follow-up, 95.7% showed no signs of hemorrhoidal disease upon examination [2]. Another study reported an overall success rate of 97.3% for THD in cases of hemorrhoidal disease at grades II, III, and IV without ischemic changes, both as a primary intervention and on subsequent procedures [5].

Although the incidence of complications associated with THD surgery is relatively low, the presence of complications should be recognized. Common complications include bleeding, pain, infection, urinary retention, and anal stricture. There is no specific incidence rate reported for horseshoe abscess after THD. However, the perianal abscess is a known complication of THD, with an incidence rate of 0.8% [6].

Horseshoe abscesses typically initiate in the deep posterior anal space, situated deep to the external sphincter and inferior to the levator ani muscle, advancing into either the unilateral or bilateral ischiorectal spaces [3]. Effectively draining the deep posterior anal space is pivotal in managing horseshoe abscesses. The primary approach involves making an incision to the ischiorectal space and a counter incision on one or both sides to facilitate drainage of any infection extension from the postanal space into the ischiorectal spaces. This method ensures comprehensive drainage and is fundamental in the treatment of horseshoe abscesses.

It is important to note that the incidence of complications may vary among studies and may depend on factors such as the severity of the hemorrhoids and the experience of the surgeon. Therefore, physicians should be aware of this potential complication and closely monitor patients for signs of infection or abscess formation after THD surgery.

## Conclusions

THD is an effective treatment for internal hemorrhoids, but horseshoe abscesses can occur in high-risk patients. Surgeons should be aware of the possibility of abscess formation following THD and consider this diagnosis in patients presenting with severe postoperative pain. Early recognition and treatment can prevent serious complications.
